# Effects of Neuromodulation on Excitatory–Inhibitory Neural Network Dynamics Depend on Network Connectivity Structure

**DOI:** 10.1007/s00332-017-9438-6

**Published:** 2018-01-04

**Authors:** Scott Rich, Michal Zochowski, Victoria Booth

**Affiliations:** 1 Applied and Interdisciplinary Mathematics Program, University of Michigan, Ann Arbor, MI, USA; 2 Departments of Physics and Biophysics, University of Michigan, Ann Arbor, MI, USA; 3 Departments of Mathematics and Anesthesiology, University of Michigan, Ann Arbor, MI, USA

**Keywords:** Neural networks, E–I networks, Acetylcholine, Synchrony, PING rhythms, 92C20

## Abstract

Acetylcholine (ACh), one of the brain’s most potent neuromodulators, can affect intrinsic neuron properties through blockade of an M-type potassium current. The effect of ACh on excitatory and inhibitory cells with this potassium channel modulates their membrane excitability, which in turn affects their tendency to synchronize in networks. Here, we study the resulting changes in dynamics in networks with inter-connected excitatory and inhibitory populations (E–I networks), which are ubiquitous in the brain. Utilizing biophysical models of E–I networks, we analyze how the network connectivity structure in terms of synaptic connectivity alters the influence of ACh on the generation of synchronous excitatory bursting. We investigate networks containing all combinations of excitatory and inhibitory cells with high (Type I properties) or low (Type II properties) modulatory tone. To vary network connectivity structure, we focus on the effects of the strengths of inter-connections between excitatory and inhibitory cells (E–I synapses and I–E synapses), and the strengths of intra-connections among excitatory cells (E–E synapses) and among inhibitory cells (I–I synapses). We show that the presence of ACh may or may not affect the generation of network synchrony depending on the network connectivity. Specifically, strong network inter-connectivity induces synchronous excitatory bursting regardless of the cellular propensity for synchronization, which aligns with predictions of the PING model. However, when a network’s intra-connectivity dominates its inter-connectivity, the propensity for synchrony of either inhibitory or excitatory cells can determine the generation of network-wide bursting.

## 1 Introduction

Neuromodulation of brain networks occurs via multiple pathways. The different types of modulators can wield powerful effects on neural network dynamics, as they can change intrinsic firing properties of neurons as well as alter their effective synaptic strengths. While anatomical synaptic connectivity of a neural network plays a primary role in dictating neural activity patterns, the ultimate dynamics exhibited by a network depends critically on which of many neuromodulators are acting on it (Bargmann and Marder 2013; Marder 2012).

One of the brain’s most potent neuromodulators is acetylcholine (ACh). In the brain, ACh levels change across sleep and wake states, with high levels during waking contributing to arousal, attention, memory and motivation. ACh affects intrinsic neuronal properties as well as synaptic transmission through two major pathways: nicotinic and muscarinic receptors. In individual neurons, ACh blocks the slow, potassium-mediated M-type ionic current via muscarinic channels, which has a threefold effect on cellular properties: (1) altering the current–frequency (I–F) curve, to increase excitability, (2) increasing spike frequency adaptation (SFA) and 3) altering neuronal phase response curves (PRCs) (Aton et al. 2013; Ruivo et al. 2013; Ermentrout et al. 2001; Stiefel et al. 2008). In cortical and hippocampal networks, excitatory pyramidal cells and some types of inhibitory interneurons can contain the M-type potassium current and thus are targets for these effects of ACh (Stiefel et al. 2008; Saraga et al. 2003; Lawrence et al. 2006; Perrenoud et al. 2013; Markram et al. 2004).

The PRC is an experimentally obtainable measure that characterizes membrane excitability properties. Namely, it measures changes in neuronal response to small membrane potential perturbations and can serve as an indicator of neuronal propensity for synchronization to network activity (Ermentrout 1996; Ermentrout et al. 2001; Schultheiss et al. 2014). Blockade of the M-current can switch the neuronal PRC from a Type II to a Type I profile. Generally, Type I neurons are characterized by a steep I–F curve with arbitrarily low firing frequency at firing threshold and by a PRC exhibiting only phase advances in response to a brief, excitatory current pulse. In contrast, Type II neurons are characterized by a more shallow I–F curve with a minimum firing frequency at threshold, and a PRC that exhibits phase delays in response to a brief, excitatory current pulse early in the neuron’s firing cycle (Wang 2010). It has been shown that Type II neurons have a higher propensity for synchronization than Type I neurons in excitatory networks (Ermentrout 1996; Hansel et al. 1995), while these neurons may synchronize via different mechanisms within inhibitory networks (Rich et al. 2016).

Given the paramount effect cholinergic modulation has on intrinsic cellular properties and a neuron’s tendency to exhibit synchrony, here we investigate its influence on the synchronous dynamics of networks of inter-connected excitatory and inhibitory neurons (E–I networks), as such networks are ubiquitous in the brain (Brea et al. 2009; Kopell et al. 2010; Best et al. 2007). The interactions of excitatory and inhibitory neurons can generate oscillatory bursts of synchronous spiking of excitatory cells which underlie rhythmic electrical activity observed in electroencephalogram (EEG) recordings associated with different brain states and cognition (Cannon et al. 2014; Whittington et al. 1995).

Computational modeling has played a major role in identifying the properties of E–I networks required to robustly generate such rhythms, including the conceptual model for rhythm generation known as Pyramidal Interneuron Network Gamma (PING) (Traub et al. 1997; Kopell et al. 2010; Whittington et al. 2000; Ermentrout and Kopell 1998) as well as other studies in which rhythms with sparse firing of the excitatory population are modeled (Hoseini and Wessel 2016). PING rhythmicity requires strong inter-connectivity between the excitatory and inhibitory subpopulations so that activity in the excitatory population quickly elicits a corresponding burst of activity in the inhibitory population, which then rapidly suppresses firing in the excitatory population. Through this reciprocal interaction, inhibition quells additional excitatory cell firing following the inhibitory burst, promoting synchronous excitatory activity after the inhibitory signal dissipates. Strong intra-connectivity within the inhibitory cell population also plays a role in the PING mechanism by ensuring that the inhibitory population fires a single synchronous burst following excitatory input. While this intra-connectivity has an abundance of biological motivation (Ferguson et al. 2013; Karson et al. 2009; Tateno and Robinson 2007; Perrenoud et al. 2013; Markram et al. 2004; Mody and Pearce 2004), strong intra-connectivity is not strictly necessary for PING-like rhythms to arise (Rich et al. 2017), making inter-connectivity between excitatory and inhibitory neurons the paramount aspect of network connectivity underlying PING.

In this paper, we analyze how the connectivity of E–I networks affects the influence of cholinergic modulation on the generation of synchronous excitatory bursting. Utilizing computational simulations of E–I networks with neurons modeled in the Hodgkin–Huxley formalism, we simulate the effect of ACh by blocking an M-type potassium current in our neuron models. We investigate all four combinations of modulatory tone of excitatory and inhibitory cells, namely excitatory or inhibitory cells with high (Type I) or low (Type II) modulatory tone. We note that the cases of mixed networks, where one subpopulation exhibits Type I properties and the other exhibits Type II properties, could conceivably arise if cholinergic release is nonuniform or if either subpopulation exhibited the given properties and lacked muscarinic receptors. To vary network connectivity structure, we focus on the effects of the strengths of inter-connections between excitatory and inhibitory cells (E–I synapses and I–E synapses), and the strengths of intra-connections among excitatory cells (E–E synapses) and among inhibitory cells (I–I synapses). Our results show that depending on the network connectivity, neuromodulation that changes the cellular propensity for synchronization may or may not affect the generation of network synchrony.

## 2 Methods

### 2.1 Neuron Models

The E–I networks studied here are comprised of neurons modeled, in the Hodgkin–Huxley formalism, on the cortical pyramidal neuron (Fink et al. 2011; Stiefel et al. 2009). This neuron is modulated by ACh such that it can display either Type I or Type II properties, and thus allows us to analyze the role of neuromodulation in these networks. The equations governing this model are

(1) $$\frac{dV}{\;dt}=-g_{Na}m_{\infty }^{3}h\left(V-E_{Na}\right)\;-g_{K_{d}}n^{4}\left(V-E_{K}\right)-g_{K_{s}}z\left(V-E_{K}\right)-g_{L}\left(V-E_{L}\right)+I_{app}-I_{syn}$$

(2) $$\frac{dX}{\;dt}=\frac{X_{\infty }(V)-X}{\tau_{X}(V)}\text{for}\;X=h,n,z$$

(3) $$m_{\infty }(V)=\frac{1}{1+e^{\left(-V-30/9.5\right)}}$$

(4) $$h_{\infty }(V)=\frac{1}{1+e^{\left(V+53/7.0\right)}}$$

(5) $$n_{\infty }(V)=\frac{1}{1+e^{\left(-V-30/10\right)}}$$

(6) $$z_{\infty }(V)=\frac{1}{1+e^{\left(-V-39/5\right)}}$$

(7) $$\tau_{h}(V)=0.37+\frac{2.78}{1+e^{(V+40.5)/6}}$$

(8) $$\tau_{n}(V)=0.37+\frac{1.85}{1+e^{(V+27)/15}}$$

(9) $$\tau_{z}(V)=75$$

V represents the membrane voltage in [mV], while m,n,h and z represent the unitless gating variables of the ionic current conductances. $I_{\text{app}}$ signifies the external applied current to the neuron (described below), in $\left[\mu A/{cm}^{2}\right]$, while $I_{\text{syn}}$ describes the synaptic current input to the cell from the network (described below), also with units of $\left[\mu A/{cm}^{2}\right].E_{Na},E_{K_{s}},E_{K_{d}}$ and $E_{L}$ are the reversal potentials and $g_{Na},g_{K_{s}},g_{K_{d}}$ and $g_{L}$ are the maximum conductances, with Na symbolizing sodium, K symbolizing potassium, and L symbolizing the leak current. K_d_ refers to the delayed rectifier potassium current, while K_s_ refers to the slow M-type potassium current. In this model, the reversal potentials are $E_{Na}=55mV,E_{K}=-90mV,E_{L}=-60mV$, while the maximum conductances are $g_{Na}=24mS/{cm}^{2},g_{K_{d}}=3mS/{cm}^{2}$, and $g_{L}=0.02mS/{cm}^{2}$.

**Fig. 1:**
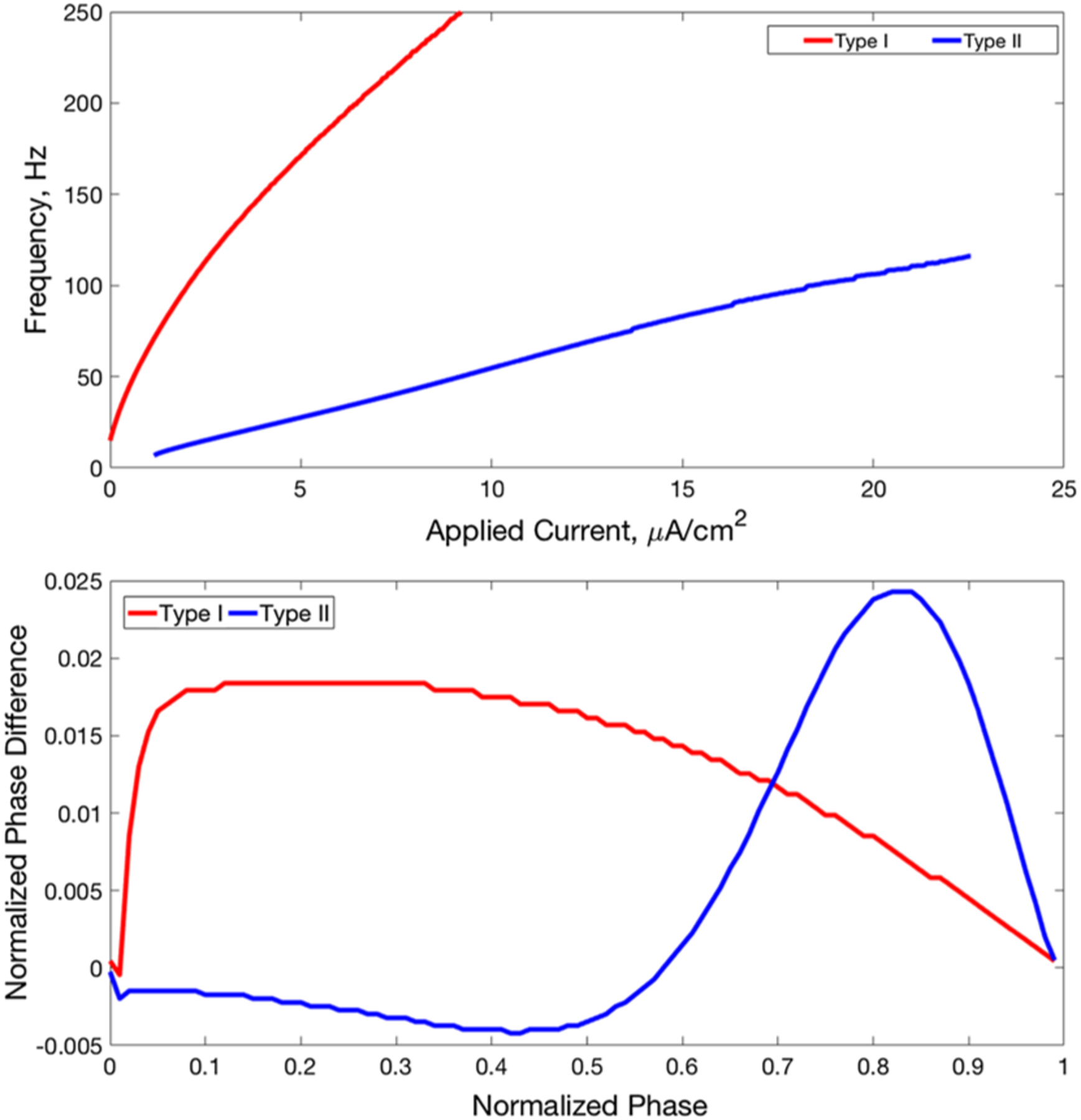
Properties of Type I and Type II neuron models **a** I–F curves for Type I (red) and Type II (blue) cells. **b** PRCs for Type I (red) and Type II (blue) cells

The conductance $g_{K_{s}}$ dictates whether the neuron displays Type I or Type II properties. We consider two values of this term: when $g_{K_{s}}=0mS/{cm}^{2}$, the neuron displays Type I properties (biologically, due to complete blockade of the slow M-type potassium channel by ACh), and when $g_{K_{s}}=1.5mS/{cm}^{2}$, the neuron displays Type II properties (biologically, due to the absence of ACh that permits maximal activity of the slow M-type potassium channel). These properties are illustrated by the I–F curves and PRCs displayed in Fig. 1. We note that the only difference between the Type I and Type II neuron models is the activity, or lack thereof, of the M-type potassium channel; thus, the M-current, and the corresponding cholinergic modulation, is entirely responsible for the differences between the Type I and Type II neuron properties. The range of $g_{K_{s}}$ values used here have been shown to replicate experimentally measured ACh-induced changes in I–F and PRC curves in cortical pyramidal neurons (Stiefel et al. 2008, 2009). We note that in this study we model only the effect of ACh on this M-current, and not any of the other potential modulatory effects of ACh.

We also emphasize that this model is used for both the excitatory and inhibitory neurons in this study. While these equations were initially developed to model the excitatory cortical pyramidal neuron, the properties of this neuron when $g_{K_{s}}=0mS/{cm}^{2}$ closely mirror those of fast-spiking Type I inhibitory interneurons, such as the parvalbumin positive (PV+) interneurons (Ferguson et al. 2013), as well as interneurons containing an M-current blocked by ACh. When the inhibitory interneurons are modeled as Type II with $g_{K_{s}}=1.5mS/{cm}^{2}$, their properties mirror those of interneurons like the oriens-lacunosum moleculare (OLM) and somatostatin-expressing (SOM) cells, which exhibit an active M-current when ACh concentrations are low (Saraga et al. 2003; Lawrence et al. 2006; Cutsuridis and Hasselmo 2012; Cutsuridis et al. 2010; Perrenoud et al. 2013; Markram et al. 2004). These similarities justify our use of equations that were originally developed to model excitatory cells for inhibitory cells as well.

### 2.2 Network Structure

Our E–I networks consist of 1000 neurons, 800 excitatory and 200 inhibitory. Excitatory neurons receive an external driving current (described below) and also receive inhibition from the inhibitory cells, where each inhibitory cell has a 50% chance to synapse onto a given excitatory cell. Inhibitory neurons receive a external current (described below) depending upon their cell type in order to ensure they do not fire in the absence of excitatory input and are near their firing threshold. Inhibitory neurons are driven by the excitatory cell population, as each excitatory cell has a 50% chance to synapse onto a given inhibitory cell. Additionally, both the inhibitory and excitatory neurons have a 30% probability of synapsing onto neurons within their subpopulation, forming the intra-connectivity of the network. The choice of this connectivity density is motivated by evidence for this level of intra-connectivity among interneurons in the hippocampus (Viriyopase et al. 2016; Ascoli and Atkeson 2005), and this connectivity density is matched among the excitatory neurons for consistency.

Cell heterogeneity was implemented by varying the external input current, $I_{app}$, to each excitatory neuron. The range of input currents were chosen such that the intrinsic firing frequencies of neurons had a range of 10 Hz. In the results displayed below, the average intrinsic cell firing frequency for an excitatory cell is 50 Hz, meaning that the minimum current, $I_{{\text{app}}_{max}}$, is the current that would cause an isolated neuron to fire at 45 Hz, while the maximum current, $I_{{\text{app}}_{max}}$, is the current that would cause an isolated neuron to fire at 55 Hz. The currents are then chosen from a uniform distribution of the form $U\left(I_{{\text{app}}_{\text{min}}},I_{{\text{app}}_{\text{max}}}\right)$.

Inhibitory neurons receive an external current to ensure they will not fire without excitatory input and are near firing threshold. Type I inhibitory cells were given a small external hyperpolarizing current to ensure that the neurons would not fire spontaneously, given that this neuron model exhibits spontaneous firing with no external current. Type II inhibitory cells were given a small depolarizing current such that these neurons were closer to their firing threshold. Variability was implemented in these currents to impart mild heterogeneity to the inhibitory population: the external current for each interneuron was chosen uniformly from the distribution $U\left(.95I_{A},1.05I_{A}\right)$, where $I_{A}$ is the average current. $I_{A}=-0.2mS/{cm}^{2}$ for Type I inhibitory cells, and $I_{A}=1.0mS/{cm}^{2}$ for Type II inhibitory cells.

We modeled synapses using a double exponential profile of the form

(10) $$I_{syn}(t)=g_{syn}\left(V-E_{\text{syn}}\right)\left(\sum_{s_{i}}\;\;e^{-\left(t-s_{i}\right)/\tau_{d}}-e^{-\left(t-s_{i}\right)/\tau_{r}}\right)$$

where $g_{\text{syn}}$ is the maximum conductance, V is the membrane voltage of the postsynaptic neuron, $E_{\text{syn}}$ is the reversal potential of the synaptic current, $s_{i}$ are the times of all presynaptic spikes occurring before the current time t in ms and $\tau_{d}$ and $\tau_{r}$ are the synaptic decay and the synaptic rise time constants, respectively (in ms). $E_{\text{syn}}$ is set at −75 mV for inhibitory synapses and 0 mV for excitatory synapses. $\tau_{r}$ is set at 0.2 ms for all synapses, while $\tau_{d}$ is set at 3.0 s for excitatory synapses and 5.5 ms for inhibitory synapses.

We alter the structure of our E–I networks by varying the strength of the various synapses (E–E, E–I, I–I, I–E) in the network. This is done by varying the maximum conductances ($g_{\text{syn}}$) for the corresponding type of synapse. To analyze the role of interconnectivity (E–I, I–E) versus intra-connectivity (E–E, I–I), the inter-connectivity strengths are varied jointly, as are the intra-connectivity strengths. Since in our networks there are four times as many excitatory cells than inhibitory cells, the E–E synaptic conductance values in the network are 1/4 of the I–I synaptic conductance values shown in the horizontal axis of Figs. 3, 4 and 6.

**Fig. 2:**
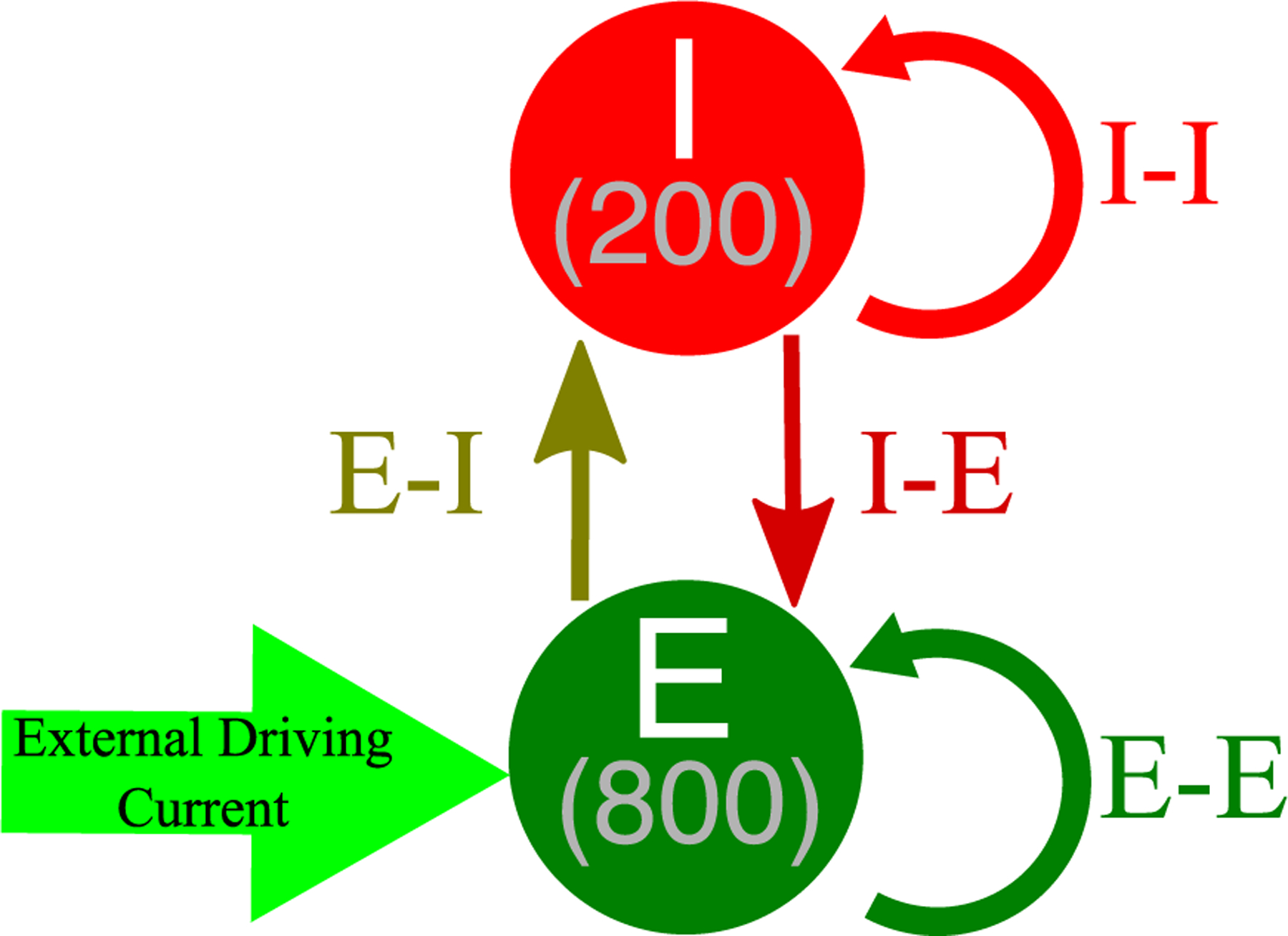
Network diagram for E–I networks. Network connectivity for E–I networks used for all simulations performed in this work. The various synaptic strengths (E–E, I–I, E–I, I–E) are altered in order to change the network connectivity structure

The connectivity diagram of the E–I networks studied here is shown in Fig 2.

### 2.3 Measures

We used several measures to quantify the dynamics of network activity. Foremost among them is the Synchrony Measure, an adaptation of a measure created by Golomb and Rinzel (1993, 1994) that quantifies the degree of spiking coincidence in the network. Briefly, the measure involves convolving a Gaussian function with the time of each action potential for every cell to generate functions $V_{i}(t)$. The population averaged voltage V(t) is then defined as $V(t)=\frac{1}{N}\sum_{i=1}^{N}\;V_{i}(t)$, where N is the number of cells in the network. We further define the overall variance of the population averaged voltage σ and the variance of an individual neuron’s voltage $\sigma_{i}$ as

(11) $$\sigma =\left\langle V(t{)}^{2}\right\rangle -\langle V(t)\rangle^{2}$$

and

(12) $$\sigma_{i}=\left\langle V_{i}(t{)}^{2}\right\rangle -{\left\langle V_{i}(t)\right\rangle }^{2}$$

where ⟨⋅⟩ indicates time averaging over the interval for which the measure is taken. The Synchrony Measure S is then defined as

(13) $$S=\frac{\sigma }{\frac{1}{N}\sum_{i=1}^{N}\sigma_{i}}$$

The value S=0 indicates completely asynchronous firing, while S=1 corresponds to fully synchronous network activity.

We additionally measure the burst frequency in these networks, which requires detecting instances of bursting activity. To do this, the spike times of neurons in each subnetwork are convolved with a Gaussian function to form a time trace of cumulative network activity. Each Gaussian function is of the form

(14) $$g(t)=e^{\frac{-(t-c{)}^{2}}{1.6}}$$

where c is the spike time. This trace is subsequently thresholded (with a threshold value of 40 for the excitatory population and 10 for the inhibitory population) to determine the on and off times for every burst ($b_{j}$ and $e_{j}$, respectively). We use the center of these bursts $\left(c_{j}=\frac{e_{j}+b_{j}}{2}\right)$ to calculate the average burst frequency by determining the time between subsequent bursts $\left(c_{j+1}-c_{j}\right)$, averaging these values, and then converting into frequency.

### 2.4 Simulations

The code implementing these simulations was written in the C programming language and run on the University of Michigan’s Flux cluster, a Linux-based high-performance computing cluster.

All simulations were run for 1500 ms from random initial conditions for voltage and gating variables for each neuron. Possible initial conditions for V ranged between −62 and −22mv, while the possible initial conditions for the gating variables n and h ranged between 0.2 and 0.8, while the initial conditions for the gating variable z ranged between 0.15 and 0.25.

Model equations were integrated using a fourth order Runge–Kutta technique. Spikes do not trigger synaptic current until 100 ms into the simulation to allow initial transients to decay.

Example raster plots shown throughout this paper are plotted such that the excitatory cells with the highest external driving current are given the lowest Neuron Index and thus are plotted toward the bottom of the y-axis, while neurons with lowest external driving current are given the highest Neuron Index and thus are plotted toward the top of the y-axis. This fashion of organizing the excitatory cells is chosen in order to more clearly illustrate the organization of these cells within a burst and does not reflect their location in the network.

All plots illustrating the various measures used to quantify network dynamics display the average of these scores over five independent simulations, where the measures are calculated over the last second of the simulation.

## 3 Results

**Fig. 3:**
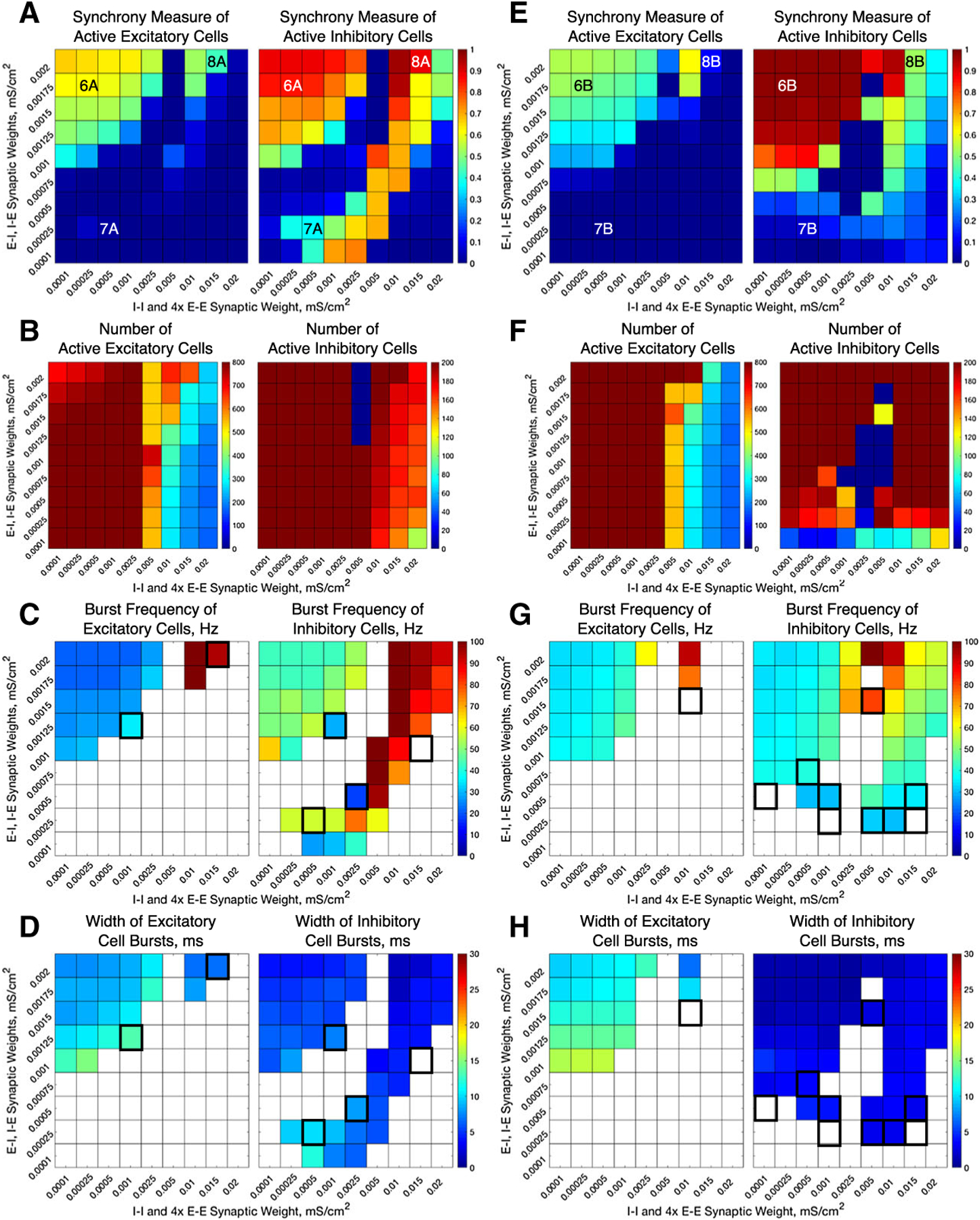
E–I networks with Type I excitatory cells primarily exhibit bursting dynamics of the excitatory subpopulation when the network inter-connectivity dominates network intra-connectivity. Spatiotemporal dynamics for E–I networks with Type I excitatory cells and Type I inhibitory cells (**a**–**d**) or with Type II inhibitory cells **e**–**g** as E–E and I–I intra-connectivity strength (x axis) and E–I and I–E inter-connectivity strength (y axis) is varied. Dynamics are quantified by the degree of synchrony for active cells (**a**, **e**), number of active cells (**b**, **f**), the burst frequency (**c**, **g**), and the burst width (**d**, **h**), where results for excitatory cells are shown in the left panels and results for inhibitory cells are shown in the right panels. Overlaid alphanumeric codes on **a** and **d** indicate simulations for which an example raster plot is shown in the indicated figure. **c**, **d**, **g** and **h** display values (i.e., nonwhite coloring) only for networks for which the burst detection mechanism identified repetitive bursting for a majority of the simulations, and the value plotted is the average only of networks when repetitive bursting was detected. Networks in which bursting is detected in three or four of the five repetitions run have their colored entry surrounded by a bolded outline. Networks in which bursting is detected in only one or two of the five repetitions run have their white entry surrounded by a bolded outline

**Fig. 4:**
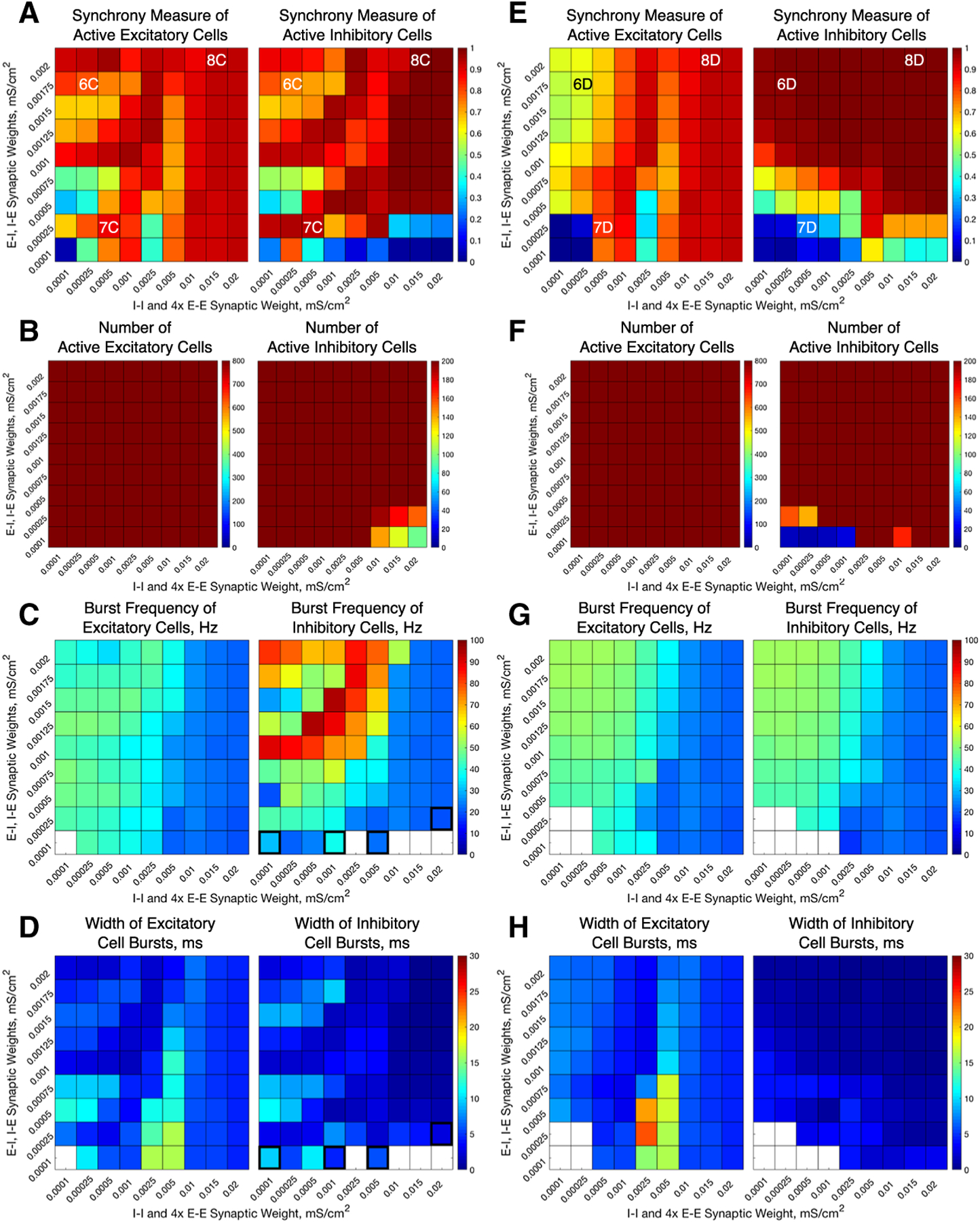
E–I networks with Type II excitatory cells can exhibit bursting dynamics of the excitatory subpopulation not just when network inter-connectivity dominates network intra-connectivity, but also in other parameter regimes driven by the propensity of Type II excitatory cells to synchronize via excitatory signaling. Spatiotemporal dynamics for E–I networks with Type II excitatory cells and Type I inhibitory cells (**a**–**d**) or with Type II inhibitory cells **e**–**g** as E–E and I–I intra-connectivity strength (x axis) and E-I and I–E inter-connectivity strength (y axis) is varied. Dynamics are quantified by the degree of synchrony for active cells (**a, e**), number of active cells (**b**, **f**), the burst frequency (**c**, **g**), and the burst width (**d**, **h**), where results for excitatory cells are shown in the left panels and results for inhibitory cells are shown in the right panels. Overlaid alphanumeric codes on **a** and **d** indicate simulations for which an example raster plot is shown in the indicated figure. **c**, **d**, **g**, **h** display values (i.e., nonwhite coloring) only for networks for which the burst detection mechanism identified repetitive bursting for a majority of the simulations, and the value plotted is the average only of networks when repetitive bursting was detected. Networks in which bursting is detected in three or four of the five repetitions run have their colored entry surrounded by a bolded outline. Networks in which bursting is detected in only one or two of the five repetitions run have their white entry surrounded by a bolded outline

Our computational study of E–I networks reveals that network dynamics depend jointly on the network connectivity structure, namely the relative strength of inter- and intra-connectivity between and within the excitatory and inhibitory subnetworks, and the neuromodulation of excitatory and inhibitory cells. We consider ACh’s effect on both excitatory and inhibitory cells that switches neuronal response properties, as measured by the PRC and I–F curves, from Type II to Type I, thus affecting the cellular propensity of synchronization. Considering the possibility of nonuniform cholinergic release or that excitatory and inhibitory cells exhibit Type I or Type II properties without the presence of an M-current, we investigate all four combinations of excitatory and inhibitory cells, namely excitatory or inhibitory cells with high (Type I) or low (Type II) modulatory tone. To vary network connectivity structure, we focus on the effects of the strengths of inter-connections between excitatory and inhibitory cells (E–I synapses and I–E synapses), and the strengths of intra-connections among excitatory cells (E–E synapses) and among inhibitory cells (I–I synapses). Figures 3 and 4 show measures of network dynamics as E–E and I–I intra-connectivity strengths are varied together (horizontal axes) and E–I and I–E inter-connectivity strengths are varied together (vertical axes) for networks with Type I excitatory cells (Fig. 3) and Type II excitatory cells (Fig. 4), and inhibitory cells exhibiting either Type I or Type II properties. Network dynamics roughly divide into three parameter regions in which synchronous bursting of the excitatory cells is differentially affected by a combination of the modulation of cellular properties and network connectivity structure.

### 3.1 High E–I and I–E Inter-connectivity Promotes Synchronous Excitatory Bursting Regardless of Cellular Properties

**Fig. 5:**
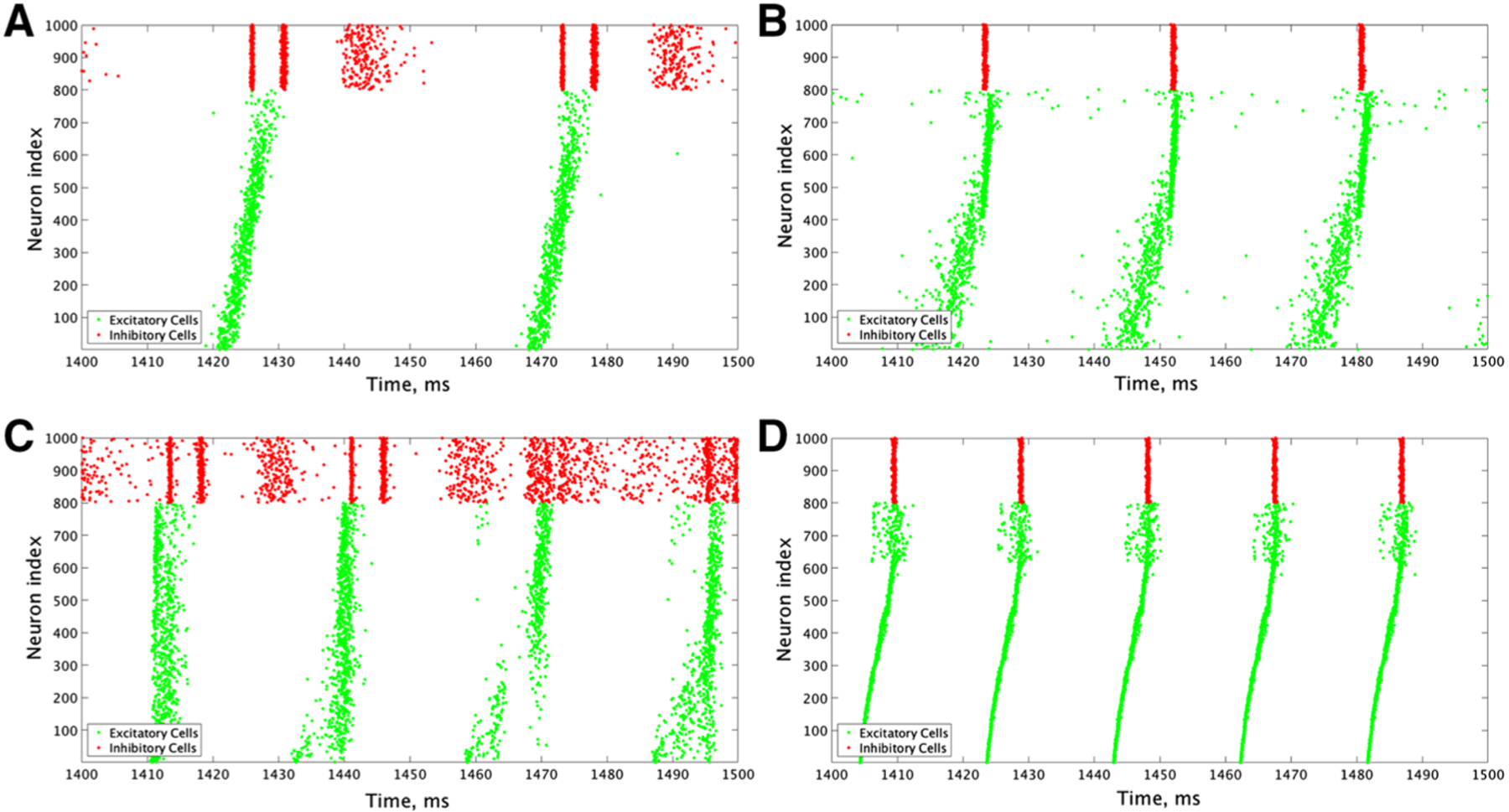
Raster plots from an example network where inter-connectivity dominates intra-connectivity illustrate synchronous excitatory cell dynamics for all combinations of cell types, albeit with varying profiles of the excitatory bursting and inhibitory dynamics. a–d Example raster plots from a network with an E–I and I–E connectivity strength of 0.00175 mS/cm^2^, an I–I connectivity strength of 0.00025 mS/cm^2^ and an E–E connectivity strength of 0.0000625 mS/cm^2^. a is **a** network with Type I excitatory and inhibitory cells, **b** is a network with Type I excitatory and Type II inhibitory cells, **c** is a network with Type II excitatory and Type I inhibitory cells, and **d** is a network with Type II excitatory and inhibitory cells. In each case, synchronous patterns are apparent in the excitatory network, although the bursting patterns exhibited by the inhibitory cells vary depending on their cell type

When E–I and I–E inter-connectivity strength dominates over E–E and I–I intra-connectivity strength (upper-left corners of heatmaps in Figs. 3, 4), all networks exhibit synchronous excitatory bursting regardless of the cellular propensity for synchrony modulated by ACh. Values of the Synchrony Measure (panels a and e) are high in this parameter regime for all modulatory conditions, with networks with Type II excitatory cells (Fig. 4) reporting higher values due to more coincident spike firing predicted by their cellular properties. Synchronous activity is robust with approximately all cells participating in the activity bursts (panels b and f), and the frequency of bursts is similar in all networks (panels c and g). Furthermore, the widths of both the excitatory and inhibitory bursts remain narrow in this regime, with narrower excitatory bursts exhibited by networks with Type II excitatory cells due to the additional synchrony promoted by excitatory intra-connectivity and the properties of Type II PRCs (Figs. 3, 4d, h). We note that the detection of excitatory bursts was robust to repeated simulations of these networks, with only one set of network connectivities (whose position in the heatmap is identified by the bolded outline in Fig. 3c, d for which bursts are detected in some, but not all, of the simulations run. As shown in the raster plots in Fig. 5, whether excitatory cells are Type I (Fig. 5a, b) or Type II (Fig. 5c, d) they fire in synchronous bursts. Thus, for this network connectivity, neuromodulation of cellular propensity for synchronization has little effect on the generation of excitatory bursting.

The synchronous excitatory bursting in these networks with high inter-connectivity is predicted and governed largely by the PING mechanism. In the PING mechanism, the inhibitory cells serve to “silence” the excitatory cells following an inhibitory burst, which causes all of the excitatory cells to return to the same point of their oscillatory firing cycle and subsequently fire synchronously when released from inhibition (Traub et al. 1997; Kopell et al. 2010; Whittington et al. 2000; Ermentrout and Kopell 1998). Evidence of the PING mechanism at work lies in the occurrence of inhibitory cell synchronous bursts near the end of excitatory cell synchronous bursts or immediately following these bursts, and in the effective silencing of excitatory activity by the inhibitory burst.

**Fig. 6:**
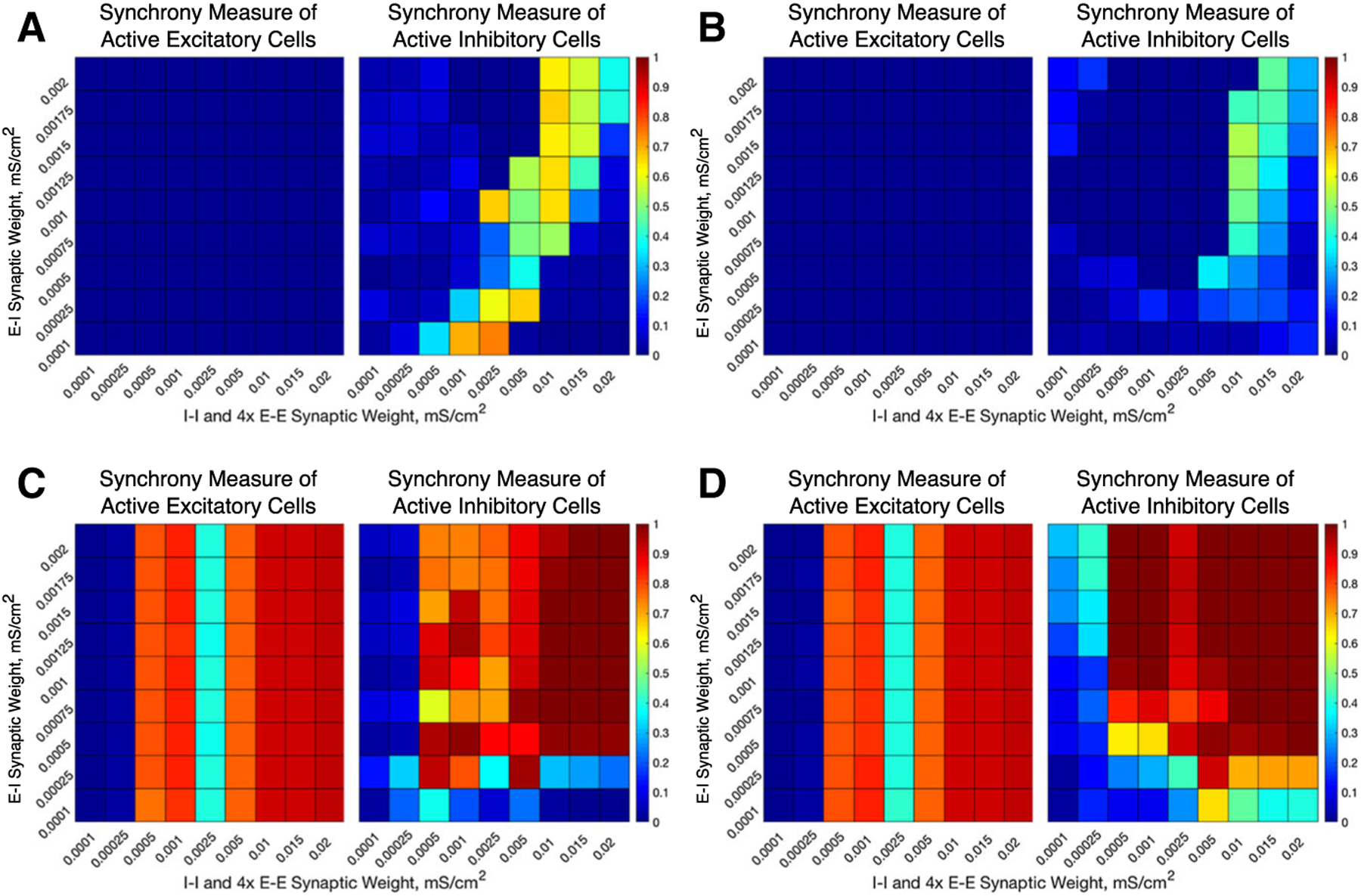
E–I networks with Type I excitatory cells cannot exhibit excitatory subpopulation synchrony in the absence of I–E connectivity, while this connectivity is not necessary to elicit excitatory subpopulation synchrony in most cases when the excitatory cells are Type II. a–d Heatmaps illustrating the degree of synchrony achieved by excitatory (left panel) and inhibitory (right panel) populations in E–I networks without any I–E connectivity for each combination of excitatory and inhibitory cell type. Such networks with Type I excitatory cells, shown in a, b, exhibit no synchrony in the excitatory cell population, implying that the excitatory bursting patterns achieved in networks with strong inter-connectivity in Fig. 3a, d are driven by inhibitory signaling to the excitatory population. In contrast, networks with Type II excitatory cells, shown in **c**, **d**, still exhibit excitatory synchrony for a majority of networks (excepting those with the lowest degree of intra-connectivity). The similarities between the parameter regimes exhibiting excitatory synchrony here and in Fig. 4a, d, where I–E connectivity was active, imply that excitatory intra-connectivity rather than network inter-connectivity may drive synchronous excitatory subpopulation dynamics in these networks

For additional verification, we simulated the same networks, but with all synaptic connections from the inhibitory cells to the excitatory cells (I–E synapses) removed (Fig. 6). In the parameter region discussed above, no synchronous activity is obtained when excitatory cells are Type I, confirming that synchronous inhibitory signaling is necessary to induce excitatory synchronous bursting in these networks. When excitatory cells are Type II, synchronous activity emerges as intra-connectivity strength increases in this parameter regime, as expected from the propensity for Type II neurons to synchronize in response to excitatory connectivity. However, obtaining synchrony for the weakest intra-connectivity strength values, as obtained in the networks containing I–E synapses, depends critically on inhibitory signaling.

In these networks, cellular properties influence the patterning of spike firing within the PING-driven synchrony. The patterning of inhibitory cell activity in response to an excitatory burst relies heavily on the inhibitory cell type, which in turn can cause subtle changes in excitatory cell dynamics. When the excitatory cells are Type I, this effect is primarily seen through the burst frequencies of the excitatory and inhibitory subpopulations (Fig. 3c, g). Comparing the example raster plot with Type I excitatory cells and Type I inhibitory cells (Fig. 5a) to that with Type I excitatory cells and Type II inhibitory cells (Fig. 5b), we see that the slower excitatory burst frequency of the former network is due to the multiple bursts of inhibitory activity in response to a burst of excitatory activity, which provides a longer lasting inhibitory synaptic signal to the excitatory cells. In contrast, when the inhibitory cells are Type II, only one instance of inhibitory activity follows excitatory activity, allowing the excitatory cells quicker release from this inhibitory signal.

The role of inhibitory cell patterning when the excitatory cells are Type II is seen primarily via differences in the Synchrony Measure and is shown by comparing example networks with Type I inhibitory cells (Fig. 5c) to those with Type II inhibitory cells (Fig. 5d). In the latter case, each excitatory burst elicits a single inhibitory burst of activity including all inhibitory cells, which ensures each excitatory cell receives a near-identical profile of inhibitory synaptic current. This allows the excitatory cells to organize based upon their external driving current, with cells with the highest external current firing earliest in the burst and those with the lowest firing latest. This organization causes the burst to occur over a longer time interval, slightly lowering the Synchrony Measure. However, when the inhibitory cells are Type I, we again see them respond to an instance of excitatory network bursting with multiple instances of activity; importantly, in this case the profile of these bursts varies in response to different instances of excitatory activity due to the randomness in the connectivity of the network as well as the heterogeneity in external drive to the cells. Variations in the inhibitory activity cause a disparity in the inhibitory signal felt by each excitatory cell, which disrupts organization in the excitatory bursts. However, this disorder also allows the burst to occur over a shorter timescale, increasing the Synchrony Measure. Thus, the inhibitory cell type plays a key role in explaining the slight difference in the Synchrony Measure seen in these networks when comparing Fig. 4a, e, while a negligible effect is seen in the frequency of the bursts (Fig. 4c, g).

### 3.2 Cellular Properties Dictate Synchronous Excitatory Bursting When E–E and I–I Intra-connectivity is High

**Fig. 7:**
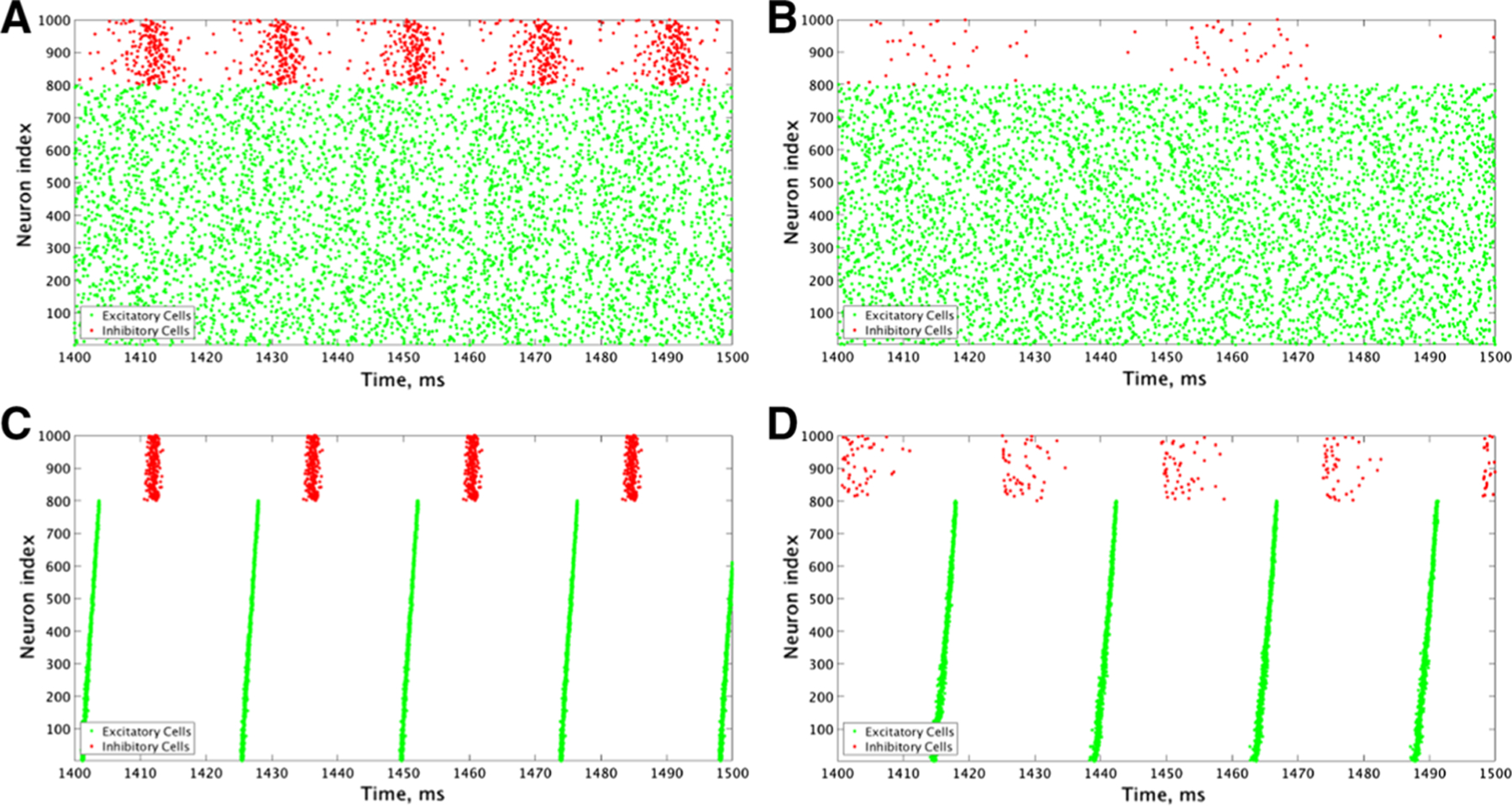
Raster plots from an example network with low inter-connectivity and slightly higher intra-connectivity illustrate that networks with Type I excitatory cells cannot achieve synchronous bursting dynamics, while networks with Type II excitatory cells can. **a–d** Example raster plots from a network with an E–I and I–E connectivity strength of 0.00025 mS/cm^2^, an I–I connectivity strength of 0.0005 mS/cm^2^ and an E–E connectivity strength of 0.000125 mS/cm^2^. **a** is a network with Type I excitatory and inhibitory cells, **b** is a network with Type I excitatory and Type II inhibitory cells, **c** is a network with Type II excitatory and Type I inhibitory cells, and **d** is a network with Type II excitatory and inhibitory cells. Only networks with Type II excitatory cells can achieve excitatory synchrony, although inhibitory synchrony is achieved without excitatory synchrony in (**A**)

When E–E and I–I intra-connectivity dominates over E–I and I–E inter-connectivity, obtaining synchronous excitatory bursting depends on the cellular propensity for synchrony. For the ranges of synaptic strengths considered here, this parameter regime begins when intra-connectivity strength is slightly higher than inter-connectivity strength (near 7a, b labels in Figs. 3, 7c, d labels in Fig. 4). When intra-connectivity is much higher than inter-connectivity (lower-right corners of heatmaps in Figs. 3, 4), the high I–I synaptic strength acts to slow firing of the inhibitory cells to the point that they cannot fire synchronously, thus minimizing their influence on excitatory subnetwork dynamics. In this regime, networks with Type I excitatory cells have Synchrony Measure values close to zero for excitatory cells (Fig. 3a, e left panels) reflecting asynchronous firing (as shown in the examples in Fig. 7a, b), as predicted by their cellular properties. While the majority of excitatory cells are firing (Fig. 3b, f left panels), no synchronous excitatory bursts were detected, as reflected by the lack of a burst frequency value (Fig. 3c, g left panels). Networks with Type II excitatory cells, on the other hand, display synchronous excitatory bursting (as shown in the examples in Fig. 7c, d), as predicted by cellular properties, with high Synchrony Measure (Fig. 4a, d), full cell participation in synchronous bursts (Fig. 4b, e) and similar burst frequencies (Fig. 4c, g). Thus, in this network structure, ACh governs the generation of synchronous excitatory activity.

The PING mechanism is not involved in generating synchronous excitatory bursts in this parameter regime as evidenced by the long gap between the excitatory cell burst and inhibitory cell firing. Inhibitory cells fire in bursts, with higher Synchrony Measure when they are Type I (Fig. 7c, d), in response to the oscillatory excitatory signal, but inhibition is not responsible for silencing the excitatory cells after the burst since they stop firing well in advance of the inhibitory bursts. Additionally, the burst frequency is not affected by the different profiles of inhibitory firing when the inhibitory cells are Type I or Type II, reflecting their lack of influence on excitatory bursting. This is confirmed by fully removing the I–E synapses and continuing to see synchronous excitatory cell firing in this regime (Fig. 6c, d).

In this regime of dominant intra-connectivity, inhibitory cell type can play a role in the dynamics of the inhibitory cell population without significantly influencing the patterning of the excitatory subpopulation. When excitatory cells are Type I, their asynchronous firing provides a weak, nearly tonic drive to the inhibitory cells. Type I inhibitory cells with weak I–I connectivity can form synchronous patterns in response to such a drive, as shown by the example raster plot in Fig. 7a and discussed in detail in our previous work (Rich et al. 2017). In contrast, Type II inhibitory cells are less excitable and more susceptible to suppression by inhibitory signaling via the dominant intra-connectivity, preventing them from forming clearly synchronous dynamics, as shown in the example raster plot in Fig. 7b.

Meanwhile, Type II excitatory cells can synchronize driven by E–E connectivity and not network inter-connectivity as discussed above, allowing for the synchronous excitatory subpopulation dynamics shown in Fig. 7c, d. Here again, though, the type of inhibitory cell dictates the dynamics of the inhibitory subpopulation in response to this weak, but synchronous, drive to the inhibitory cells. Given that Type I inhibitory cells are more excitable, the weak burst of excitation is sufficient to prompt all of the inhibitory cells to fire in a closely clustered fashion, an example of which is shown by the raster in Fig. 7c. In contrast, properties of our Type II neuron model lead these inhibitory cells to respond with a more sparse burst to a nearly identical excitatory synaptic drive, an example of which is shown by the raster in Fig. 7d.

We note that the interaction between Type II PRC properties and strong E–E connectivity can cause some complex burst patterns in the excitatory population. This is evidenced in Fig. 4d, h by the significantly wider excitatory bursts seen in the middle of our range of network intra-connectivities. However, this behavior does not disrupt the overall oscillatory behavior of the network driven by the network intra-connectivity.

### 3.3 Cellular Properties Influence Ability of Inter-connectivity to Generate Synchronous Excitatory Bursting When Inter- and Intra-connectivity are Balanced

**Fig. 8:**
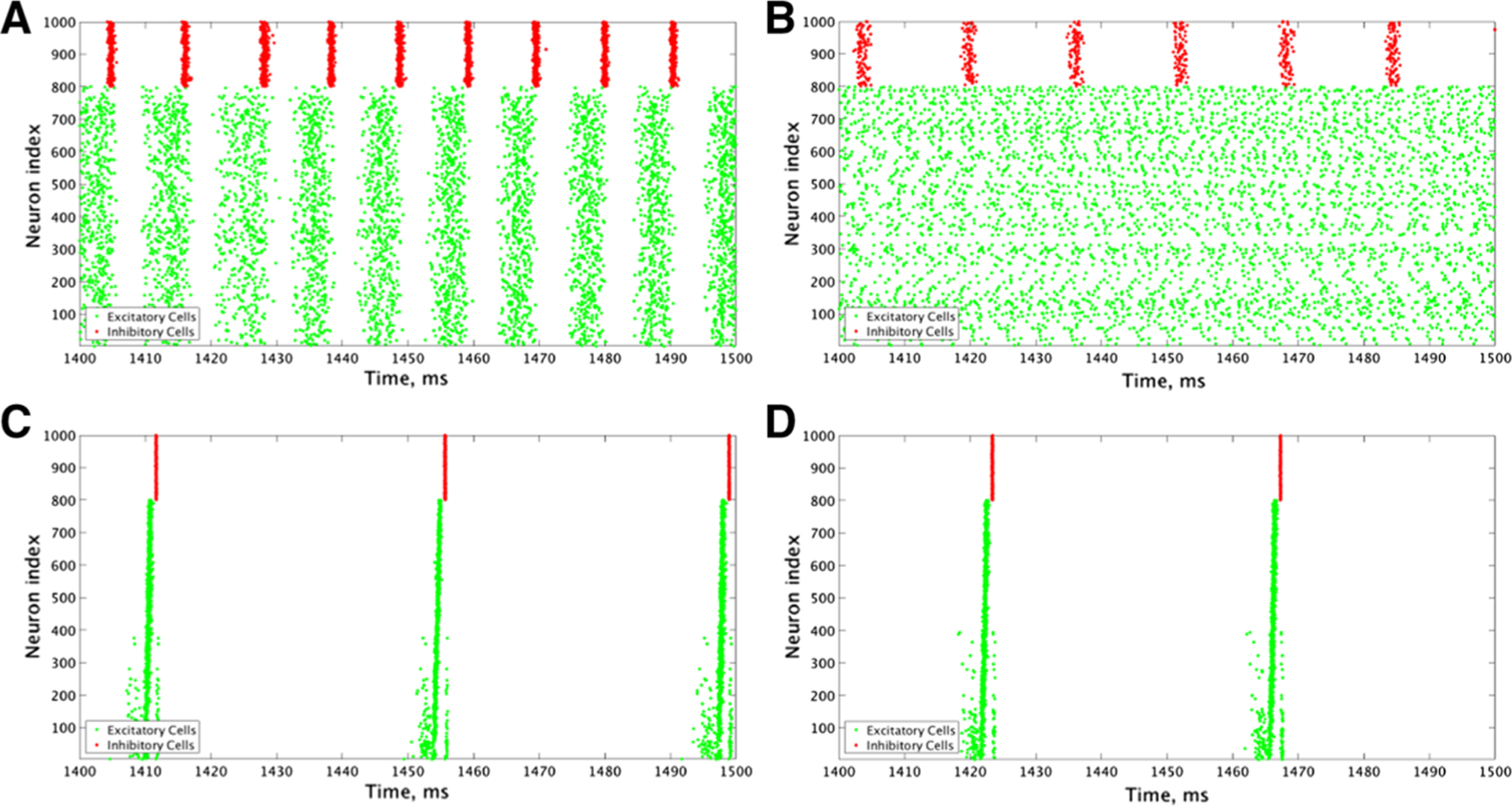
Raster plots from an example network with strong intra-connectivity and inter-connectivity reveal the tendency for high E–E connectivity to elicit depolarization block in some Type I excitatory cells, while consistent bursting patterns remain in networks with Type II excitatory cells. **a–d** Example raster plots from a network with an E–I and I–E connectivity strength of 0.002 mS/cm^2^, an I–I connectivity strength of 0.015 mS/cm^2^ and an E–E connectivity strength of 0.00375 mS/cm^2^. **a** is a network with Type I excitatory and inhibitory cells, **b** is a network with Type I excitatory and Type II inhibitory cells, **c** is a network with Type II excitatory and Type I inhibitory cells, and **d** is a network with Type II excitatory and inhibitory cells. While networks with Type II excitatory cells exhibit consistent bursting patterns in both the excitatory and inhibitory networks, networks with Type I excitatory cells have some cells shut down due to depolarization block (shown most clearly in (**B**)), which can interfere with the development of synchrony

When E–I, I–E inter-connectivity and E–E, I–I intra-connectivity are both strong in our parameter space, corresponding to the upper-right corner of the heatmaps in Figs. 3 and 4, both cellular properties and network connectivity contribute to the network’s tendency to exhibit synchronous excitatory bursting. When excitatory cells are Type I, while their cellular properties resist synchronization, loose synchronous bursting is obtained when inhibitory cells are Type I (example raster in Fig. 8a), but not Type II (example raster in Fig. 8b). This example represents one of the few instances in this parameter regime in which excitatory bursting activity was detected in some, but not all, of the simulations run (as represented by the grid squares with a bolded outline in Fig. 3c, d). As reflected in their Synchrony Measures (Fig. 3a, e), Type I inhibitory cells synchronize tightly with high I–I intra-connectivity (**A**, right panel), while Type II inhibitory cells do not (**E**, right panel). The strong inhibitory signal provided to the excitatory cells from Type I inhibitory cells silences their activity and produces a weak synchronous excitatory burst by the PING mechanism. When inhibitory cells are Type II, however, their more sparse firing has little effect on the excitatory subnetwork, and asynchronous excitatory firing persists.

When excitatory cells are Type II, their propensity for synchronization strengthens the influence of high inter-connectivity to generate robust excitatory synchronous bursting for both types of inhibitory cells (Fig. 8c, d). Indeed, Synchrony Measures for both excitatory and inhibitory subnetworks are the highest in this parameter regime (Fig. 4a, e) with full network participation in the bursts (Fig. 4b, f). Additionally, values of all measures are the same for Type I and Type II inhibitory cells. There is conflicting evidence as to whether the PING mechanism or excitatory network intra-connectivity drives this synchrony: while the inhibitory network bursts do closely follow the excitatory network bursts (Fig. 8c, d), as predicted by the PING mechanism, the removal of I–E synapses does not eliminate excitatory synchrony (Fig. 6c, d), meaning that inhibition may not serve a causal role in synchronizing excitatory cells. It is likely that some combination of these two mechanisms is what results in the strong synchrony of the excitatory network seen here.

Thus, in this network structure, cholinergic modulation acts in conjunction with high inter-connectivity to generate synchronous excitatory bursting. Synchronous excitatory bursting fails to exist only when both excitatory and inhibitory populations have a low propensity for synchronization.

In this regime of balanced inter- and intra-connectivity, cell properties contribute to the characteristics of excitatory synchronous bursting, as shown by the example raster plots in Fig. 8. For Type II excitatory cells, spikes in the synchronous bursts are highly coincident (Fig. 8c, d) due to the strong E–E intra-connectivity overpowering the heterogeneity in firing frequency that created more variation in spike timing in the low E–E intra-connectivity regime (examples in Fig. 5c, d) This induces a highly coincident burst of inhibitory cells immediately following the excitatory burst. The strong inhibitory signal to all excitatory cells coupled with their previous coincident firing leads to a long silent period between excitatory bursts and low burst frequency (Fig. 4c, g). For Type I excitatory and inhibitory cells, burst frequencies are the highest since the high E–E intra-connectivity drives the excitatory cells to recover quickly from the inhibitory signal and initiate the next excitatory burst (Fig. 3c).

The inter- and intra-connectivity strengths can also be balanced at a weak level in our parameter space, which corresponds with the lower-left corner of the heatmaps in Figs. 3 and 4. In this regime, networks with Type I excitatory cells exhibit completely asynchronous firing due to an inability to achieve PING rhythmicity, while inhibitory cells may be able to synchronize themselves due to the near-tonic drive provided by the asynchronous excitatory cells (Fig. 3a, e). Meanwhile, in networks with Type II excitatory neurons, excitatory synchrony can be achieved in networks with all, but the weakest connectivity strengths (Fig. 4a, e) driven by the ability for Type II excitatory neurons to synchronize themselves even with weak E–E synapses.

Thus, when the inter- and intra-connectivity strengths are balanced, but at weak levels, the tendency to achieve synchronous excitatory cell dynamics is controlled by ACh. In this regime, synchronous excitatory bursting occurs only due to the tendency of Type II neurons to synchronize due to E–E synapses, even when these synapses may be weak.

### 3.4 Dynamics of Inhibitory Subnetworks

Across our parameter space of network connectivity structures, there are regimes where activity in the inhibitory subnetwork does not correlate with activity in the excitatory subnetwork. These instances are largely robust to repetition, as there are few instances where inhibitory bursts are detected in some, but not all, of the repetitions for a given network (cases in which this occurs are represented the grid squares with bolded outlines in Figs. 3, 4c, d, g, h). For example, as discussed above, Type I inhibitory cells can form oscillatory synchronous bursts independent of synchronous activity in the excitatory subnetwork. This behavior is seen in our networks with Type I excitatory cells when intra-connectivity is larger than inter-connectivity (diagonal band of high Synchrony Measure in Fig. 3a, right panel). In this regime, the excitatory cells are asynchronous and provide a tonic excitatory drive to the inhibitory cells, inducing repetitive firing. When coupled with inhibitory synapses, repetitively firing Type I cells receiving strong tonic drive have a high propensity for synchronization (Rich et al. 2016). As intra-connectivity increases, maintaining synchronous bursting requires increased excitatory input to counteract the increased inhibitory signaling within the inhibitory subnetwork (resulting in the diagonal band). In fact, inhibitory activity becomes sparse and asynchronous when intra-connectivity is much higher than inter-connectivity (Fig. 3a–c right panels, lower-right corners).

In other parameter regimes, firing in the inhibitory subnetwork is almost completely suppressed. This occurs in three parameter regimes that are most easily identified in the right panels of Figs. 3 and 4b, f, which show the number of active inhibitory cells: a small region of high inter-connectivity strength for networks of Type I excitatory and inhibitory neurons (Fig. 3b), a small region with low inter-connectivity strength for networks of Type II excitatory and inhibitory neurons (Fig. 4f), and a relatively large parameter regime for networks of Type I excitatory neurons and Type II inhibitory neurons (Fig. 3f). The first two cases are easily explained. The first is a case of classic depolarization block of the Type I inhibitory neurons: the moderate intra-connectivity strength is enough to force excitatory cells to fire very quickly due to the E–E connectivity, and the high inter-connectivity strength leads these fast firing excitatory cells to provide excessive excitation to the inhibitory cell population, driving those cells into depolarization block. The second case is the opposite situation, as weak inter-connectivity combined with low excitability of Type II inhibitory cells result in insufficient excitatory synaptic signal to the inhibitory cells to induce firing.

The final case seen in networks of Type I excitatory neurons and Type II inhibitory neurons is more complex, involving intricacies of the dynamics of the M-type potassium current. The activity of this ionic current not only serves to shift the properties of the neuron PRC from Type I to Type II as discussed previously, but also imbues the neurons with spike frequency adaptation. Given the slow timescale of the z gating variable that governs this current when compared to the extremely fast timescale of the m,n and h variables governing the currents directly inciting action potential firing, the M-current acts to slow down the firing of the neuron following repetitive action potentials. Thus, the neuron “adapts” its firing frequency given the recent past, firing slower if a quick burst of action potentials occurred previously. This adaptation is reflected by a rise in the value of z as action potential firings occur. As the potassium current is a hyperpolarizing current, larger values of z that arise from action potential firing invoke a larger amplitude of the M-type potassium current which in turn slows down cell firing.

When Type II neurons are provided a tonic excitatory drive that induces repetitive firing, the adaptation current eventually settles into a stable periodic pattern that allows repetitive action potential firing at a constant frequency. However, in our E–I networks, the drive to the inhibitory population is provided by the synaptic drive from the excitatory population, which has a distinctly nontonic profile. In particular, properties of the reversal potential in the synaptic current term in Eq. 10 speed up action potential firing when compared to a tonic current with a similar maximum amplitude. This increase in firing frequency prevents z from settling into a stable oscillation, instead causing it to steadily increase. When this gating variable rises too high, the hyperpolarizing current from the M-type potassium channel exceeds the depolarizing excitatory input current from the excitatory cell population, causing a net hyperpolarizing current and in turn quiescence. In short, in certain E–I networks where the excitatory cells do not synchronize, the adaptation current prevents the inhibitory cells from exhibiting repetitive firing due to the form of the excitatory synaptic current.

## 4 Discussion

**Fig. 9:**
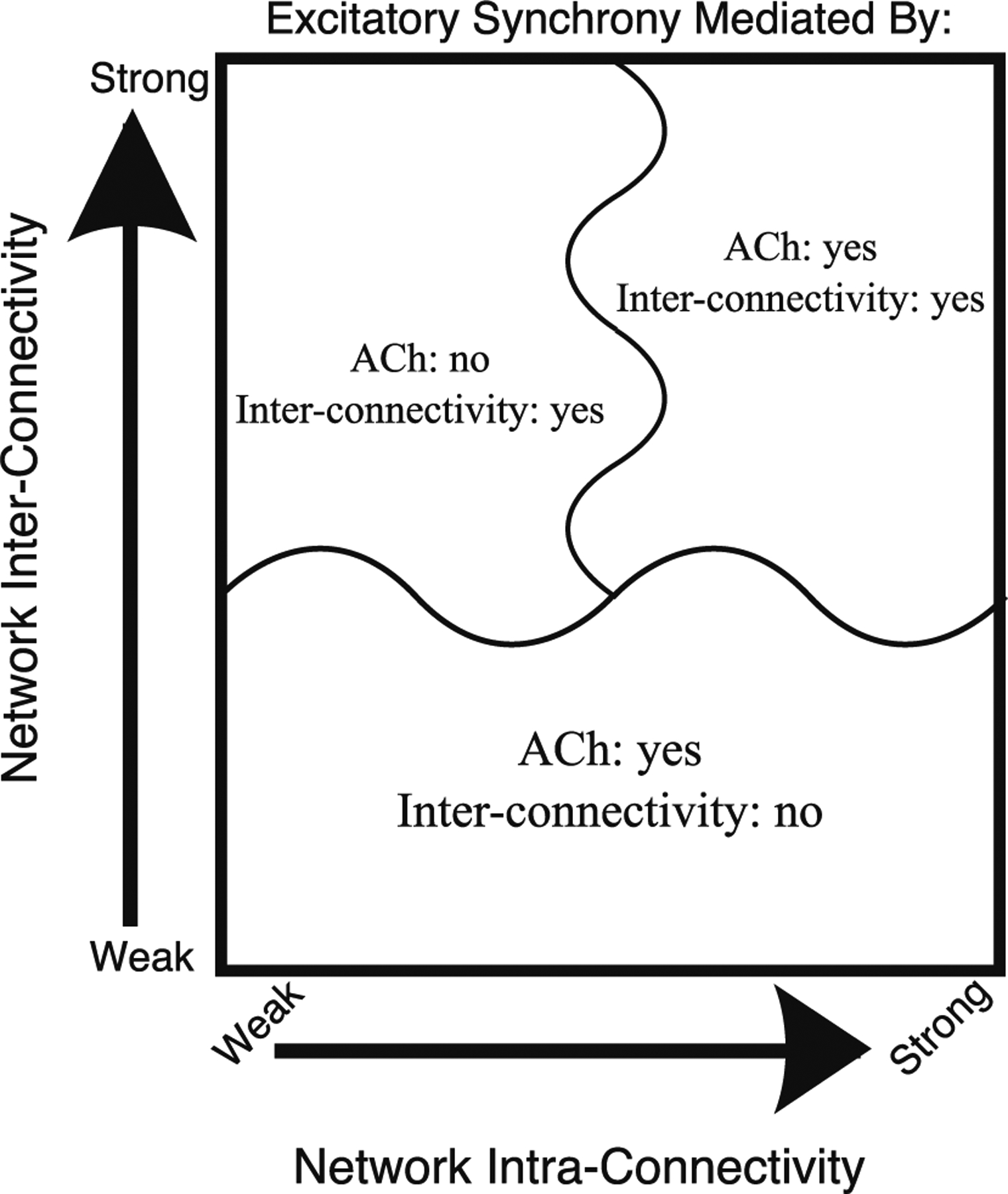
Summary figure illustrating the three regimes in which excitatory synchrony is mediated differentially by cholinergic modulation and network topology. When network inter-connectivity dominates network intra-connectivity (top-left regime), excitatory synchrony is mediated by network inter-connectivity while cholinergic modulation has minimal effect on dynamics. When network inter-connectivity is weak (bottom regime), excitatory synchrony is mediated by cholinergic modulation’s effect on cell type, and not by network inter-connectivity. Finally, when network inter- and intra-connectivity are both strong (top-right regime), cholinergic modulation and network inter-connectivity both influence the tendency for networks to exhibit excitatory synchrony, with these interactions sometimes leading to complex dynamics

Our work here reveals that the development of synchronous excitatory activity in E–I networks depends critically on both the intrinsic cellular properties of the excitatory and inhibitory cells as well as the connectivity structure of the network as described by the inter- and intra-connectivity strengths. These results are summarized in Fig. 9. In particular, depending on the network connectivity, effects of neuromodulation that change the cellular propensity for synchronization may or may not affect the generation of network synchrony. High E–I and I–E inter-connectivity that dominates over the influence of E–E and I–I intra-connectivity induces synchronous excitatory bursting regardless of the cellular propensity for synchronization. In this regime, the PING mechanism generates the excitatory bursting along with synchronous activity of inhibitory cells. When E–E and I–I intra-connectivity has a larger influence than E–I and I–E inter-connectivity, the propensity of excitatory cells to synchronize determines the generation of excitatory bursting in the networks. Even when inhibitory cells can form synchronous activity due to their cellular properties, weak inter-connectivity does not induce synchronous activity in the excitatory cells. Thus, mechanisms for PING-driven synchrony are ineffective in these network topologies.

Networks in which both inter- and intra-connectivity are strong achieve excitatory synchrony resulting from a combination of the cellular propensity for synchronization and the network inter-connectivity that drives PING. In this regime, the high propensity for synchronization of Type II excitatory cells, combined with PING-like dynamics driven by strong inter-connectivity, leads to strong synchrony irrespective of inhibitory cell properties. Meanwhile, although Type I excitatory cells resist synchrony driven by intra-connectivity, the ability for inhibitory cells to synchronize permits the PING mechanism to induce weaker synchrony in the excitatory population in some cases. Finally, networks in which both inter- and intra-connectivity are weak are able to achieve excitatory synchrony only when the excitatory cells are Type II and prone to synchronize even with weak intra-connectivity. Meanwhile, networks with Type I excitatory cells can sometimes elicit synchronous spiking of the inhibitory network due to the near-tonic drive asynchronous firing of the excitatory cells provides to the inhibitory population.

Computational studies probing oscillatory synchronous activity in E–I networks, and in particular the intricacies of PING rhythms, are prevalent in the literature (Traub et al. 1997; Kopell et al. 2010; Whittington et al. 2000; Ermentrout and Kopell 1998; Börgers and Kopell 2003; Börgers et al. 2012; Börgers and Kopell 2005; Borgers and Walker 2013; Krupa et al. 2014; Olufsen et al. 2003). These studies pay less attention to the role of intrinsic cellular properties or the impact of more varied network structures, as the conceptual PING mechanism assumes strong E–I and I–E inter-connectivity as well as strong I–I intra-connectivity and burst frequencies are presumed to be dictated by properties of synaptic currents. However, as our biological understanding of the brain rapidly accelerates, the immense diversity of neuron properties, particularly of inhibitory interneurons, and network connectivities among these interneuron populations (Brea et al. 2009; Kopell et al. 2010; Best et al. 2007; Klausberger et al. 2003; Buhl et al. 1994; Beierlein et al. 2000; Barthó et al. 2004; Somogyi and Klausberger 2005; Klausberger and Somogyi 2008; Gonchar and Burkhalter 1997; Beierlein et al. 2003; Gibson et al. 1999; Deco and Thiele 2011; Hasselmo and Giocomo 2006; Hasselmo and Sarter 2011; Sarter et al. 2005) motivates computational research to investigate and understand dynamics arising from the interaction of cellular properties and network connectivity structures.

Investigating the role of intrinsic cellular properties on E–I network dynamics through the lens of neuromodulation, particularly that achieved via the action of ACh, provides further salience to this work given the important roles this neuromodulator has in the brain. Concentrations of ACh are known to fluctuate based on sleep-wake states: ACh is present in high concentrations during wake and REM sleep, and in low concentrations during slow-wave sleep (Aton et al. 2013; Ruivo et al. 2013). Computational research has shown that changing the cholinergic modulatory tone can elicit changes in neural network dynamics that mirror those seen experimentally (Roach et al. 2015), and our work shows that the changes brought about by varying ACh concentrations are also affected by network connectivity. Furthermore, ACh also influences how the brain directs attention in response to competing stimuli (Desimone and Duncan 1995; Luck et al. 1997; Reynolds et al. 1999; Fries 2005; Bosman et al. 2012), a behavior whose corresponding neural dynamics might also be explained by analyzing the role of ACh on neural networks as done here. These hypotheses relating ACh concentration to differing neural network dynamics are supported by experiments in which direct manipulation of the M-current causes changes in network dynamics; for example, slow oscillations in excitatory networks in the motor cortex are abolished when the M-current is blocked (Castro-Alamancos et al. 2007).

More generally, however, it is clear from our study that both cellular and structural network properties are intertwined together to dictate which specific dynamical mechanisms generate observed spatiotemporal dynamics. We hypothesize that gross changes in network structure, as those observed, for example, in the epileptic brain, may lead to transitions among cellular-based and network-based dynamical mechanisms which in turn may result in transitions between cognitive and pathological brain functions (see, for example, Bharath et al. 2015; Parent et al. 1997).

## References

[R1] Ascoli GA, Atkeson JC: Incorporating anatomically realistic cellular-level connectivity in neural network models of the rat hippocampus. Biosystems 79, 173–181 (2005) 15649602 10.1016/j.biosystems.2004.09.024

[R2] Aton SJ, Broussard C, Dumoulin M, Seibt J, Watson A, Coleman T, Frank MG: Visual experience and subsequent sleep induce sequential plastic changes in putative inhibitory and excitatory cortical neurons. PNAS 110(8), 3101–3106 (2013) 23300282 10.1073/pnas.1208093110 PMC3581875

[R3] Bargmann CI, Marder E: From the connectome to brain function. Nat. Methods 10, 483–490 (2013) 23866325 10.1038/nmeth.2451

[R4] Barthó P, Hirase H, Monconduit L, Zugaro M, Harris KD, Buzsáki G: Characterization of neocortical principal cells and interneurons by network interactions and extracellular features. J. Neurophysiol 92(1), 600–608 (2004) 15056678 10.1152/jn.01170.2003

[R5] Beierlein M, Gibson JR, Connors BW: A network of electrically coupled interneurons drives synchronized inhibition in neocortex. Nat. Neurosci 3(9), 904–910 (2000) 10966621 10.1038/78809

[R6] Beierlein M, Gibson JR, Connors BW: Two dynamically distinct inhibitory networks in layer 4 of the neocortex. J. Neurophysiol 90(5), 2987–3000 (2003) 12815025 10.1152/jn.00283.2003

[R7] Best J, Park C, Terman D, Wilson C: Transitions between irregular and rhythmic firing patterns in excitatory-inhibitory neuronal networks. J. Comput. Neurosci 23(2), 217–235 (2007) 17624604 10.1007/s10827-007-0029-7

[R8] Bharath R, Sinha S, Panda R, Raghavendra K, George L, Chaitanya G, Gupta A, Satishchandra P: Seizure frequency can alter brain connectivity: evidence from resting-state fmri. Am. J. Neuroradiol 36(10), 1890–1898 (2015) 26294642 10.3174/ajnr.A4373 PMC7965029

[R9] Börgers C, Kopell N: Synchronization in networks of excitatory and inhibitory neurons with sparse, random connectivity. Neural Comput. 15(3), 509–538 (2003) 12620157 10.1162/089976603321192059

[R10] Börgers C, Kopell N: Effects of noisy drive on rhythms in networks of excitatory and inhibitory neurons. Neural Comput. 17(3), 557–608 (2005) 15802007 10.1162/0899766053019908

[R11] Borgers C, Walker B: Toggling between gamma-frequency activity and suppression of cell assemblies. Front. Comput. Neurosci 7, 33 (2013) 23596411 10.3389/fncom.2013.00033 PMC3627140

[R12] Börgers C, Franzesi GT, LeBeau FE, Boyden ES, Kopell NJ: Minimal size of cell assemblies coordinated by gamma oscillations. PLoS Comput. Biol 8(2), e1002362 (2012) 22346741 10.1371/journal.pcbi.1002362 PMC3276541

[R13] Bosman CA, Schoffelen JM, Brunet N, Oostenveld R, Bastos AM, Womelsdorf T, Rubehn B, Stieglitz T, Weerd PD, Fries P: Attentional stimulus selection through selective synchronization between monkey visual areas. Neuron 75, 875–888 (2012) 22958827 10.1016/j.neuron.2012.06.037 PMC3457649

[R14] Brea JN, Kay LM, Kopell NJ: Biophysical model for gamma rhythms in the olfactory bulb via subthreshold oscillations. Proc. Natl. Acad. Sci 106(51), 21954–21959 (2009) 19996171 10.1073/pnas.0910964106 PMC2799880

[R15] Buhl EH, Halasy K, Somogyi P: Diverse sources of hippocampal unitary inhibitory postsynaptic potentials and the number of synaptic release sites. Nature 368, 28 (1994) 10.1038/368823a0 8159242

[R16] Cannon J, McCarthy MM, Lee S, Lee J, Borgers C, Whittington M, Kopell N: Neurosystems: brain rhythms and cognitive processing. Eur. J. Neurosci 39(5), 705–719 (2014) 24329933 10.1111/ejn.12453 PMC4916881

[R17] Castro-Alamancos MA, Rigas P, Tawara-Hirata Y: Resonance (10 hz) of excitatory networks in motor cortex: effects of voltage-dependent ion channel blockers. J. Physiol 578(1), 173–191 (2007) 16945964 10.1113/jphysiol.2006.119016 PMC2075114

[R18] Cutsuridis V, Hasselmo M: Gabaergic contributions to gating, timing, and phase precession of hippocampal neuronal activity during theta oscillations. HIPPOCAMPUS 22, 1597–621 (2012) 22252986 10.1002/hipo.21002

[R19] Cutsuridis V, Cobb S, Graham P: Encoding and retrieval in a model of the hippocampal cal microcircuit. HIPPOCAMPUS 20, 423–446 (2010) 19489002 10.1002/hipo.20661

[R20] Deco G, Thiele A: Cholinergic control of cortical network interactions enables feedback-mediated attentional modulation. Eur. J. Neurosci 34(1), 146–157 (2011) 21692884 10.1111/j.1460-9568.2011.07749.x

[R21] Desimone R, Duncan J: Neural mechanisms of selective visual attention. Annu. Rev. Neurosci 18, 193–222 (1995) 7605061 10.1146/annurev.ne.18.030195.001205

[R22] Ermentrout GB: Type I membranes, phase resetting curves, and synchrony. Neural Comput. 8(5), 979–1001 (1996) 8697231 10.1162/neco.1996.8.5.979

[R23] Ermentrout GB, Kopell N: Fine structure of neural spiking and synchronization in the presence of conduction delays. Proc. Natl. Acad. Sci 95(3), 1259–1264 (1998) 9448319 10.1073/pnas.95.3.1259 PMC18738

[R24] Ermentrout B, Pascal M, Gutkin B: The effects of spike frequency adaptation and negative feedback on the synchronization of neural oscillators. Neural Comput. 13, 1285–1310 (2001) 11387047 10.1162/08997660152002861

[R25] Ferguson KA, Huh CYL, Amilhon B, Williams S, Skinner FK: Experimentally constrained ca1 fast-firing parvalbumin-positive interneuron network models exhibit sharp transitions into coherent high frequency rhthyms. Front. Comput. Neurosci 7, 144 (2013) 24155715 10.3389/fncom.2013.00144 PMC3804767

[R26] Fink CG, Booth V, Zochowski M: Cellularly-driven differences in network synchronization propensity are differentially modulated by firing frequency. PLoS Comput. Biol 7(5), e1002062 (2011) 21625571 10.1371/journal.pcbi.1002062 PMC3098201

[R27] Fries P: A mechanism for cognitive dynamics: neuronal communication through neuronal coherence. TRENDS Cognit. Sci 9(10), 474–480 (2005) 16150631 10.1016/j.tics.2005.08.011

[R28] Gibson JR, Beierlein M, Connors BW: Two networks of electrically coupled inhibitory neurons in neocortex. Nature 402(6757), 75–79 (1999) 10573419 10.1038/47035

[R29] Golomb D, Rinzel J: Dynamics of globally coupled inhibitory neurons with heterogeneity. Phys. Rev. E 48, 4810–4814 (1993) 10.1103/physreve.48.4810 9961165

[R30] Golomb D, Rinzel J: Clustering in globally coupled inhibitory neurons. Physica D 72, 259–282 (1994)

[R31] Gonchar Y, Burkhalter A: Three distinct families of gabaergic neurons in rat visual cortex. Cereb. Cortex 7(4), 347–358 (1997) 9177765 10.1093/cercor/7.4.347

[R32] Hansel D, Mato G, Meunier C: Synchrony in excitatory neural networks. Neural Comput. 7(2), 307–337 (1995) 8974733 10.1162/neco.1995.7.2.307

[R33] Hasselmo M, Giocomo L: Cholinergic modulation of cortical function. J. Mol. Neurosci 30, 133–135 (2006) 17192659 10.1385/JMN:30:1:133

[R34] Hasselmo ME, Sarter M: Modes and models of forebrain cholinergic neuromodulation of cognition. Neuropsychopharmacology 36(1), 52–73 (2011) 20668433 10.1038/npp.2010.104 PMC2992803

[R35] Hoseini MS, Wessel R: Coherent and intermittent ensemble oscillations emerge from networks of irregular spiking neurons. J. Neurophysiol 115(1), 457–469 (2016) 26561602 10.1152/jn.00578.2015 PMC4760494

[R36] Karson MA, Tang AH, Milner TA, Alger BE: Synaptic cross talk between perisomatic-targeting interneuron classes expressing cholecystokinin and parvalbumin in hippocampus. J. Neurosci 29(13), 4140–4154 (2009) 19339609 10.1523/JNEUROSCI.5264-08.2009 PMC2853357

[R37] Klausberger T, Somogyi P: Neuronal diversity and temporal dynamics: the unity of hippocampal circuit operations. Science 321(5885), 53–57 (2008) 18599766 10.1126/science.1149381 PMC4487503

[R38] Klausberger T, Magill PJ, Márton LF, Roberts JDB, Cobden PM, Buzsáki G, Somogyi P: Brain-state-and cell-type-specific firing of hippocampal interneurons in vivo. Nature 421(6925), 844–848 (2003) 12594513 10.1038/nature01374

[R39] Kopell N, Börgers C, Pervouchine D, Malerba P, Tort A: Gamma and theta rhythms in biophysical models of hippocampal circuits. In: Cutsuridis V, Graham B, Cobb S, Vida I (eds.) Hippocampal Microcircuits, pp. 423–457. Springer (2010)

[R40] Krupa M, Gielen S, Gutkin B: Adaptation and shunting inhibition leads to pyramidal/interneuron gamma with sparse firing of pyramidal cells. J. Comput. Neurosci 37(2), 357–376 (2014) 25005326 10.1007/s10827-014-0508-6

[R41] Lawrence J, Saraga F, Churchill J, Startling J, Travis K, Skinner F, McBain C: Somatodendritic kv7/kcnq/m channels control interspike interval in hippocampal interneurons. J. Neurosci 26(47), 12325–12338 (2006) 17122058 10.1523/JNEUROSCI.3521-06.2006 PMC6675427

[R42] Luck SJ, Chelazzi L, Hillyard SA, Desimone R: Neural mechanisms of spatial selective attention in areas v1, v2, and v4 of macaque visual cortex. Annu. Rev. Neurosci 77, 24–42 (1997) 10.1152/jn.1997.77.1.24 9120566

[R43] Marder E: Neuromodulation of neuronal circuits: back to the future. Neuron 76(1), 1–11 (2012) 23040802 10.1016/j.neuron.2012.09.010 PMC3482119

[R44] Markram H, Toledo-Rodriguez M, Wang Y, Gupta A, Silberberg, glad: Interneurons of the neocortical inhibitory system. Nat. Rev. Neurosci 5, 793–807 (2004) 15378039 10.1038/nrn1519

[R45] Mody I, Pearce RA: Diversity of inhibitory neurotransmission through gaba a receptors. Trends Neurosci. 27(9), 569–575 (2004) 15331240 10.1016/j.tins.2004.07.002

[R46] Olufsen MS, Whittington MA, Camperi M, Kopell N: New roles for the gamma rhythm: population tuning and preprocessing for the beta rhythm. J. Comput. Neurosci 14(1), 33–54 (2003) 12435923 10.1023/a:1021124317706

[R47] Parent JM, Timothy WY, Leibowitz RT, Geschwind DH, Sloviter RS, Lowenstein DH: Dentate granule cell neurogenesis is increased by seizures and contributes to aberrant network reorganization in the adult rat hippocampus. J. Neurosci 17(10), 3727–3738 (1997) 9133393 10.1523/JNEUROSCI.17-10-03727.1997 PMC6573703

[R48] Perrenoud Q, Rossier J, Geoffrey H, Vitalis T, Gallopin T: Diversity of gabaergic interneurons in layer via and vib of mouse barrel cortex. Cereb. Cortex 23, 423–441 (2013) 22357664 10.1093/cercor/bhs032

[R49] Reynolds JH, Chelazzi L, Desimone R: Competitive mechanisms subserve attention in macaque areas v2 and v4. J. Neurosci 19(5), 1736–1753 (1999) 10024360 10.1523/JNEUROSCI.19-05-01736.1999 PMC6782185

[R50] Rich S, Zochowski M, Booth V: Dichotomous dynamics in e-i networks with strongly and weakly intra-connected inhibitory neurons. Front. Neural Circuits (submitted, 2017) 10.3389/fncir.2017.00104 PMC5733501 29326558

[R51] Rich S, Booth V, Zochowski M: Intrinsic cellular properties and connectivity density determine variable clustering patterns in randomly connected inhibitory neural networks. Front. Neural Circuits 10, 82 (2016) 27812323 10.3389/fncir.2016.00082 PMC5071331

[R52] Roach JP, Ben-Jacob E, Sander LM, Zochowski MR: Formation and dynamics of waves in a cortical model of cholinergic modulation. PLoS Comput. Biol 11(8), e1004449 (2015) 26295587 10.1371/journal.pcbi.1004449 PMC4546669

[R53] Ruivo LMTG, Mellor JR: Cholinergic modulation of hippocampal network function. Front. Synaptic Neurosci 5, 2 (2013) 23908628 10.3389/fnsyn.2013.00002 PMC3726829

[R54] Saraga F, Wu C, Zhang L, Skinner F: Active dendrites and spike propagation in multi compartment models of oriens-lacunosum/moleculare hippocampal interneurons. J. Physiol 552, 673–689 (2003) 12923216 10.1113/jphysiol.2003.046177 PMC2343469

[R55] Sarter M, Hasselmo ME, Bruno JP, Givens B: Unraveling the attentional functions of cortical cholinergic inputs: interactions between signal-driven and cognitive modulation of signal detection. Brain Res. Rev 48(1), 98–111 (2005) 15708630 10.1016/j.brainresrev.2004.08.006

[R56] Schultheiss N, Prinz A, Butera RJ (eds.): Phase Response Curves in Neuroscience: Theory, Experiment and Analysis. Springer series in computational neuroscience. Springer, New York (2014)

[R57] Somogyi P, Klausberger T: Defined types of cortical interneurone structure space and spike timing in the hippocampus. J. Physiol 562(1), 9–26 (2005) 15539390 10.1113/jphysiol.2004.078915 PMC1665488

[R58] Stiefel KM, Gutkin BS, Sejnowski TJ: Cholinergic neuromodulation changes phase response curve shape and type in cortical pyramidal neurons. PLoS ONE 3(12), e3947 (2008) 19079601 10.1371/journal.pone.0003947 PMC2596483

[R59] Stiefel KM, Gutkin BS, Sejnowski TJ: The effects of cholinergic neuromodulation on neuronal phase-response curves of modeled cortical neurons. J. Comput. Neurosci 26(2), 289–301 (2009) 18784991 10.1007/s10827-008-0111-9 PMC2857973

[R60] Tateno T, Robinson HPC: Phase resetting curves and oscillatory stability in interneurons of rat somatosensory cortex. Biophys. J 92, 683–693 (2007) 17192317 10.1529/biophysj.106.088021 PMC1751383

[R61] Traub RD, Jefferys JG, Whittington MA: Simulation of gamma rhythms in networks of interneurons and pyramidal cells. J. Comput. Neurosci 4(2), 141–150 (1997) 9154520 10.1023/a:1008839312043

[R62] Viriyopase A, Memmesheimer RM, Gielen S: Cooperation and competition of gamma oscillation mechanisms. J. Neurophysiol 116, 232 (2016) 26912589 10.1152/jn.00493.2015 PMC4969395

[R63] Wang XJ: Neurophysiological and computational principles of cortical rhythms in cognition. Physiol. Rev 90, 1195–1268 (2010) 20664082 10.1152/physrev.00035.2008 PMC2923921

[R64] Whittington MA, Traub RD, Jefferys JG: Synchronized oscillations in interneuron networks driven by metabotropic glutamate receptor activation. Nature 373(6515), 612 (1995) 7854418 10.1038/373612a0

[R65] Whittington M, Traub RD, Kopell N, Ermentrout B, Buhl E: Inhibition-based rhythms: experimental and mathematical observations on network dynamics. Int. J. Psychophysiol 38, 315–336 (2000) 11102670 10.1016/s0167-8760(00)00173-2

